# Demethylation of the TSDR Is a Marker of Squamous Cell Carcinoma in Transplant Recipients

**DOI:** 10.1111/ajt.12899

**Published:** 2014-09-23

**Authors:** S N Sherston, K Vogt, S Schlickeiser, B Sawitzki, P N Harden, K J Wood

**Affiliations:** 1 Transplantation Research Immunology Group, Nuffield Department of Surgical Sciences, University of OxfordOxford, United Kingdom; 2 Institute for Medical Immunology, Charité–University MedicineBerlin, Germany; 3 BCRT Berlin Brandenburg Center for Regenerative Therapies, Charite University MedicineBerlin, Germany; 4 Oxford Transplant Centre, Churchill HospitalOxford, United Kingdom

## Abstract

Malignancy is an important cause of death in transplant recipients. Cutaneous squamous cell carcinoma (cSCC) causes significant morbidity and mortality as 30% of transplant recipients will develop cSCC within 10 years of transplantation. Previously we have shown that high numbers of regulatory T cells (Tregs) are associated with the development of cSCC in kidney transplant recipients (KTRs). Demethylation analysis of the Treg-specific demethylated region (TSDR) provides a more accurate association with cSCC risk after transplantation. Age, gender and duration of immunosuppression matched KTRs with (n = 32) and without (n = 27) cSCC, were re-analyzed for putative clinical and immunological markers of cancer risk. The proportion of FOXP3+ CD4+ cells was higher in the population with a previous SCC. Major T cell subsets remained stable over time; although B cell, CD8 and CD4 subpopulations demonstrated age-related changes. TSDR methylation analysis allowed clarification of Treg numbers, enhancing the association of high Treg levels in KTRs with cSCC compared to the cSCC-free cohort. These data validate and expand on previous findings in long-term KTRs, and show that immune markers remain stable over time. TSDR demethylation analysis provides a more accurate biomarker of cancer posttransplantation.

## Introduction

Transplantation is the treatment of choice for individuals with end-stage kidney failure, with current 1-year graft survival rates greater than 90% (1). Unfortunately, good short-term outcomes have not been accompanied by improvements in long-term graft and patient survival (2). Side effects of nonspecific immunosuppression can result in nephrotoxicity and significant morbidity and mortality from cardiovascular disease (3), infection (4) and malignancy (5). Morbidity and mortality from malignancy is an increasing problem and is now the commonest cause of death in transplant recipients in Australia and New Zealand (6). Furthermore, 10-year incidence of malignancy in UK organ transplant recipients is twice the rate of the general population (7). Although there is substantial variation in the standardized incidence ratio of different cancers posttransplantation, the greatest prevalence is seen in cutaneous squamous cell carcinoma (cSCC) (7)–(9). Organ transplant recipients are 200 times more likely to develop a cSCC than the general population (10). cSCC grow more rapidly and metastasize more readily in kidney transplant recipients (KTRs) resulting in increased morbidity and mortality (11,12). Mechanisms underlying the substantial increase in cSCC prevalence posttransplantation include skin type, ultra-violet light exposure, chronic human papilloma virus colonization and type and duration of immunosuppression (11). The impact of immunosuppression suggests that modulation of the immune system plays an important role in the development of cancers posttransplantation. Analysis of immune cell subsets to identify individuals at increased risk of cancer posttransplantation has proved informative. Low CD4 lymphocyte counts may predict cancer risk (13), while other studies have focused on immune-regulatory or effector cell populations. Increased numbers of regulatory T cells (Tregs) in the peripheral circulation is associated with a poor prognosis in cancer in the general population (14,15). Previously we have shown that high numbers of Treg (FOXP3^+^CD4^+^CD25^+^CD127^low^) and low numbers of natural killer (NK) cells are associated with an increased risk of developing cSCC posttransplantation (11). Tregs were characterized by the expression of FOXP3, but subsequently it has become clear that FOXP3 may also be expressed by activated nonregulatory human T cells (16,17). Recently, a Treg-specific demethylated region (TSDR) in the FOXP3 locus was identified that is associated with stable FOXP3 expression, allowing accurate quantification of naturally occurring (n)Treg alone (18).

In this study, we have investigated the hypothesis that an increased risk of developing cSCC after transplantation is associated with high numbers of FOXP3^+^CD4^+^CD127^low^ TSDR-demethylated bona fide Treg. We have retested survivors of a cohort of long-term KTRs, with and without skin cancer, to determine whether the peripheral blood immune phenotype remains stable over time and to identify TSDR-demethylated cells. We investigated the frequency of nTreg in peripheral blood by epigenetic analysis of the TSDR, and explored the prognostic value of CD4^+^ TSDR-demethylated cells as a marker of Treg associated with cSCC development.

## Concise Methods

### Patients

The study re-recruited KTRs with and without histologically diagnosed cSCC who had been previously matched for age (±5 years), gender and time on immunosuppression (±5 years). Patients with second or subsequent grafts had the duration of immunosuppression summed. KTRs without cSCC could have cSCC *in situ* (Bowen's disease), basal carcinoma or keratoacanthomas. KTRs with skin types I–IV were selected because cSCC predominantly affects fair-skinned populations.

**Figure 1:**
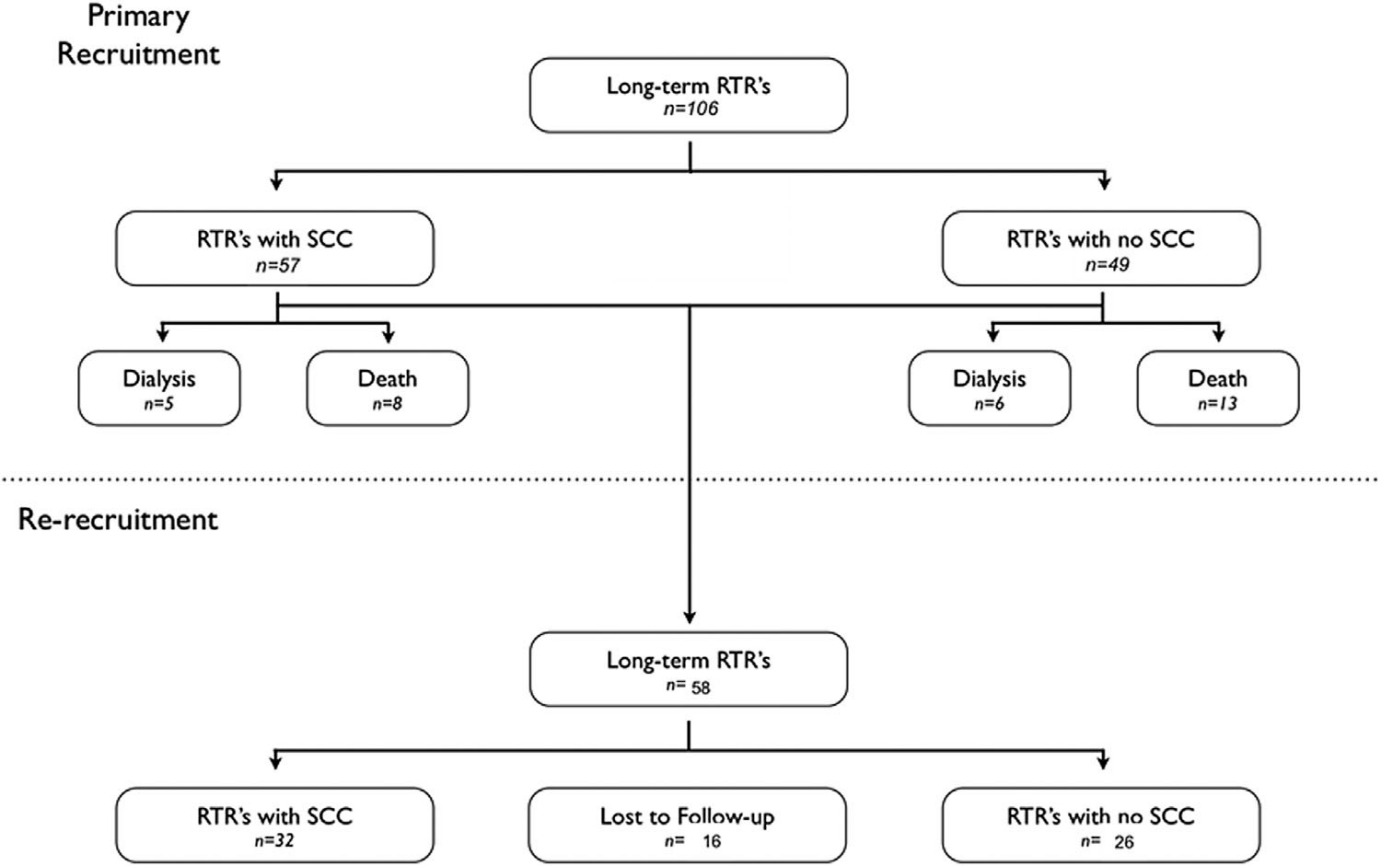
Flow diagram showing recruitment of long-term KTR with (n = 57) and without (n = 49) a previous SCC into the initial study. Subsequently all available KTRs (n = 58) from the primary study were re-recruited for repeat immunophenotyping (median of 1595 days [1135–2025] later). (Exclusions: 11 [10.4%] graft failures; 21 [19.8%] deaths; 16 [15.1%] lost to follow-up.) Causes of death included: 9 (42.9%) cardiovascular; 7 (33.3%) malignancy; and 5 (23.8%) from infection. KTR, kidney transplant recipient; SCC, cutaneous squamous cell carcinoma.

All 106 transplant patients previously recruited were invited for re-recruitment, 74 of these patients (69.8%) were identified as still alive with a functioning graft (Figure 1). The study was approved by a multi-center ethics committee and performed according to Strengthening the Reporting of Observational Studies in Epidemiology (STROBE) guidelines for observational studies. Written informed consent was obtained from all patients who participated. Three KTRs declined to be assessed, eight KTRs had been transferred to hospitals away from the Oxford University Hospitals NHS Trust and five KTRs we were lost to follow-up. At the end of re-recruitment in March 2013, 32 KTRs with SCC and 26 KTRs without SCC had been re-recruited. Case records and the hospital histopathology database were reviewed, and demographic, transplantation and histopathologic variables were recorded. Tumors that were defined as re-excision or as arising from scar tissue were excluded.

### Immune phenotyping

Peripheral blood was collected from KTRs during at routine clinical follow-up. Patients were asked about current skin lesions to ensure blood was not taken during active disease. Peripheral blood mononuclear cells (PBMCs) were separated from whole blood by standard centrifugation and Ficoll techniques within a median of 40 min (range 30–100 min) of venesection. Lymphocytes were stained as described previously and analyzed via flow cytometry (11). Absolute counts for cell populations identified were calculated using total lymphocyte count from routine hematology laboratory results and the proportion of each cell type in the lymph gate from flow cytometry data. Immune phenotyping was postponed when the following were present: fever >37.8°C or oral or intravenous antibiotic or antiviral therapy current or completed with 2 weeks. Patients who were admitted 1 week after immune phenotyping for suspected or confirmed infection were re-assessed during a clinically quiescent period.

### DNA methylation analysis of the TSDR

Freshly isolated PBMCs were surface stained for CD3 and CD4 to identify the number of lymphocytes CD3^+^CD4^+^. Genomic DNA from whole isolated PBMCs was extracted with the QIAamp DNA blood mini kit (Qiagen, Hilden, Germany). A minimum of 60 ng bisulfite-treated (EpiTect; Qiagen) genomic DNA was used in a real-time polymerase chain reaction (PCR) to quantify the FOXP3 TSDR. Real-time PCR was performed in a final reaction volume of 20 µL containing 10 µL FastStart universal probe master (Roche Diagnostics, Mannheim, Germany), 50 ng/µL lamda DNA (New England Biolabs, Frankfurt, Germany), 5 pmol/µL methylation or nonmethylation-specific probe, 30 pmol/µL methylation or nonmethylation-specific primers and 60 ng bisulfite-treated DNA or a respective amount of plasmid standard. The samples were analyzed in triplicate on an ABI 7500 cycler (Life Technologies Ltd, Carlsbad, CA).

### Statistical analysis

All statistical analysis was performed using the statistical software SPSS 16 (SPSS, Chicago, IL) and GraphPad Prism 5 (GraphPad Software, San Diego, CA). A value of p < 0.05 was considered significant in all statistical tests.

## Results

A total of 58 (55.7% of original study cohort) KTRs were recruited (n = 32 KTRs with cSCC; n = 26 without cSCC). The mean follow-up time for all patients re-recruited was 1595.1 days (1135–2025) since initial immune phenotype analysis. In the SCC cohort 73% developed a further cSCC during the follow-up period, with 50% developing more than one cSCC. In contrast only 4% of the non-cSCC cohort developed a cSCC during the same period. A total of 21 (19.8%) patients had died since initial recruitment (Figure 1). Furthermore 11 KTRs (10.4%) had lost their graft and 16 (15.1%) were lost to follow-up (Figure 1). The group with and without cSCC remained well matched for age, sex, number of transplants, HLA mismatches and duration of immunosuppression (Table1). We found no significant difference between the type of immunosuppression between the cSCC and non-cSCC cohorts, although four of the cSCC group were on an mTOR inhibitor, and three of the four had been maintained on an mTOR inhibitor throughout the study period. The mean (±SD; range) Cyclosporin trough level at time of second sampling was 79 ng/mL (±27; 30–148): mean Sirolimus trough level 4.8 ng/mL (±1.0; <3–5.6): mean Tacrolimus trough level 7.4 ng/mL (±4.0; 4.6–12).

**Table 1:** Clinical and immunological characteristics of long-term KTRs with and without cSCC

	SCC (n = 32)	No SCC (n = 26)	p-Value
Age at assessment, years (mean [range])	68.5 (53–87)	64.9 (42–78)	p = 0.16
Age at first transplant, years (mean [range])	47.1 (21–66)	44.1 (18–63)	p = 0.36
Number of transplants (mean [range])	1.4 (1–3)	12 (1–3)	p = 0.54
Male gender (N [%])	28 (87.5%)	21 (80.8%)	p = 0.48
Years of immunosuppression (mean [range])	20.9 (11–48)	20.3 (6–31)	p = 0.76
Current azathioprine use (N [%])	19 (59.4%)	13 (50%)	p = 0.48
Current CycA use (N [%])	19 (59.4%)	16 (61.5%)	p = 0.87
Curent Tac use (N [%])	2 (6.3%)	1 (3.8%)	p = 0.68
Current prednisolone use (N [%])	12 (37.5%)	6 (23.1%)	p = 0.24
Creatinine, μmol/L (median [range])	134 (67–348)	127 (83–264)	p = 0.26
HLA mismatch (mean [range])	2.4 (0–6)	2.6 (0–5)	p = 0.61
% CD4+ cells FOXP3+ (median [range])	4.7 (1.1–12.5)	3.4 (1.4–8.3)	p = 0.017
CD4+ FOXP3+ cells/μL (median [range])	21.9 (3–101)	19.3 (8–612)	p = 0.29
% CD8+ CD28− (median [range])	53.5 (7.9–88.8)	61.7 (9.1–94.2)	p = 0.83
CD4+ cells/μL (median [range])	580.2 (85–2168)	633.4 (215–1282)	p = 0.64
CD8+ cells/μL (median [range])	307.2 (28–1069)	370.9 (75–921)	p = 0.10
% Central memory CD8+ (median [range])	2.1 (0.3–11)	2.1 (0.2–15.8)	p = 0.77
NK cells/μL (median [range])	73.4 (1–702)	22.2 (2–615)	p = 0.10

cSCC, cutaneous squamous cell carcinoma; CycA, Cyclosporin A; KTR, kidney transplant recipient; NK, natural killer; Tac, Tacrolimus.

**Figure 2:**
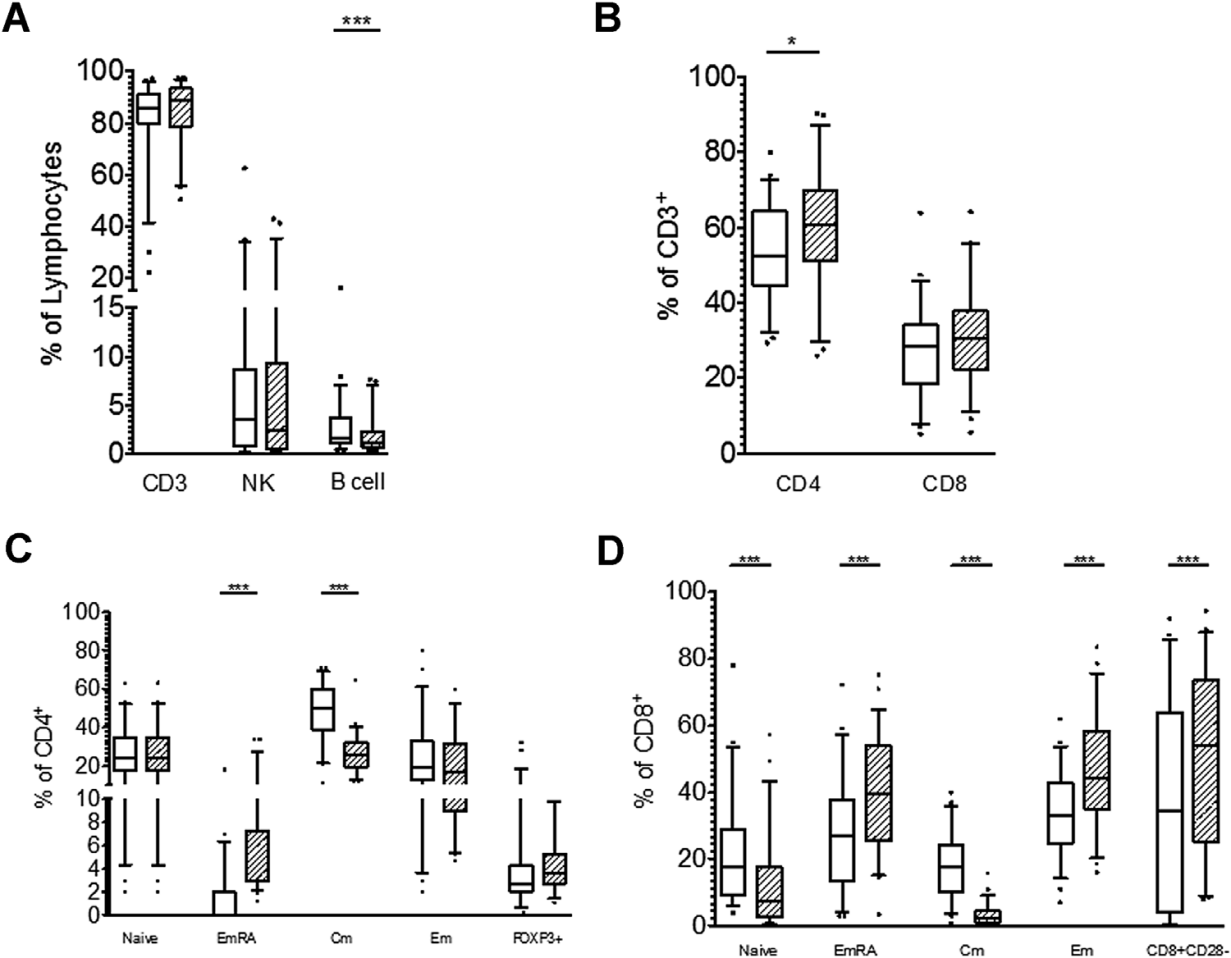
Box plots showing original (open box) and current (cross-hatched box) lymphocyte subset data from immunophenotyping of re-recruited kidney transplant recipients. (A and B) Box plots showing stability of major lymphocyte subsets. No significant changes were observed in the proportion of T cells (CD3^+^), natural killer cells (CD3− CD56+ CD16+) or CD8+ T cells. A minor but significant increase in the proportion of CD4+ cells was observed. A large and significant decrease in the proportion of B cells occurred in the patients over the follow-up period. CD4+ (C) and CD8+ (D) T cell subsets show significant changes in many of the compartments. The proportion of FOXP3+ CD4+ T cells remains stable over the follow-up period. ***p < 0.001, *p = 0.03.

**Figure 3:**
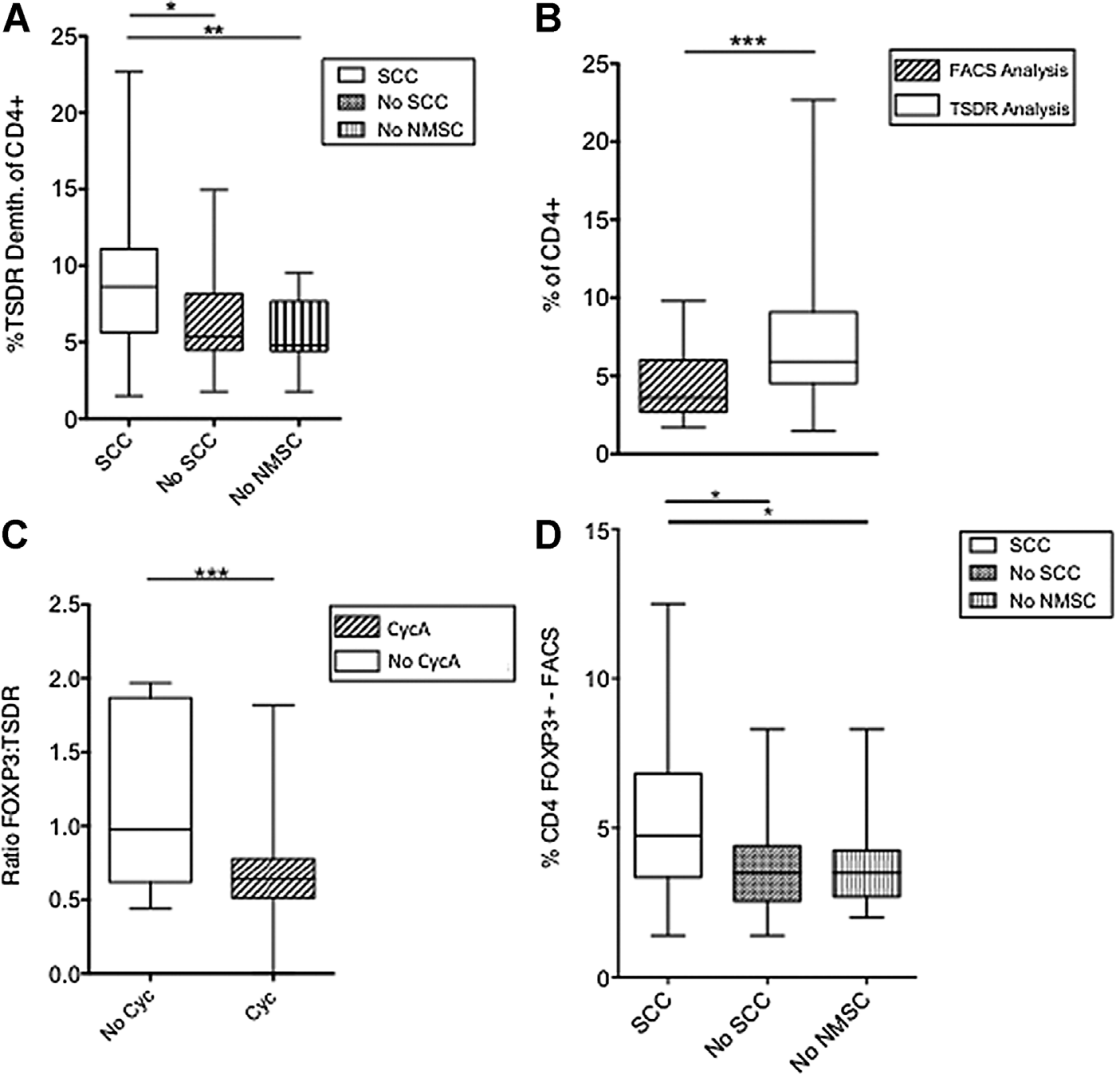
(A) Box plot shows the percentage of CD4+ lymphocytes that are TSDR demethylated in patients who have (open box) or have not (cross-hatch box) developed an SCC posttransplant. KTRs who have never developed an NMSC are shown in the vertical striped box. Patients who have not previously developed an SCC have a significantly lower proportion of TSDR-demethylated CD4+ cells than those who have developed an SCC. Patients who have had no type of NMSC have even more significantly less TSDR-demethylated CD4+ cells than patients who have had a previous SCC. (B) Box plot showing the percentage of CD4+ cells that are FOXP3+, are significantly lower than the proportion of TSDR-demethylated CD4+ cells (p < 0.001). (C) Box plot showing the ratio of FOXP3+ to TSDR-demethylated CD4+ lymphocytes in KTRs currently treated with (cross-hatch box) and without (open box) the calcineurin inhibitor (CNI), Cyclosporin A (CycA). The majority of patients who had Cyc A in their drug regimen had FOXP3/TSDR ratios of <1, while those who were on CNI-free regimens were around 1. (D) Box plot showing the percentage of CD4+ lymphocytes analyzed by fluorescence-activated cell sorting that are FOXP3+ in patients who have (open box) or have not (cross-hatch box) developed an SCC post transplant. KTRs who have never developed an NMSC are shown in the vertical striped box. Patients who have not previously developed an SCC or any NMSC have a significantly lower proportion of FOXP3+ CD4+ cells than those who have developed an SCC. KTR, kidney transplant recipient; NMSC, nonmelanoma skin cancer; SCC, squamous cell carcinoma; TSDR, Treg-specific demethylated region.

We found a significant increase in the proportion of FOXP3+ CD4+ T cells with a previous cSCC (Figure 3D). Second, we assessed the stability of the main lymphocyte subsets in the current cohort and compared the data with that from matched patients in the original cohort (Figure 2). We found no differences in the proportion of CD8 or NK cells between the two groups. There was a small but significant rise in the number of CD4+ cells over the follow-up period (p = 0.03), and a significant reduction in the proportion of B cells (p = 0.006). In the analysis of CD4+ and CD8+ lymphocyte subgroups we found age-typical changes in the proportions of naïve (p < 0.001), central memory (Cm; p < 0.001), effector memory (Em; p < 0.001), effector memory RA (EmRA; p < 0.001) and CD8+ CD28− regulatory cells (p < 0.001) (19). We observed a nonsignificant rise in the proportion of CD4+ FOXP3+ cells.

The utilization of the methylation status of the TSDR region to identify true Tregs identified a significant increase in the proportion of CD4+ TSDR-demethylated cells in patients who had previously developed an cSCC (p = 0.03; Figure 3). KTRs who had never developed a nonmelanoma skin cancer had a significantly lower number of TSDR-demethylated CD4+ cells than those who have had a previous cSCC (p = 0.008). Interestingly, the proportion of CD4+ lymphocytes that were FOXP3+ was significantly less than the proportion of TSDR-demethylated CD4+ cells. Moreover, the ratio of FOXP3+/TSDR-demethylated CD4+ lymphocytes was significantly lower in patients who had Cyclosporin A in their drug regimen compared with those who were calcineurin inhibitor (CNI) free (p < 0.001; Figure 3). We explored the potential predictive value of FOXP3+ CD4+ T cells and CD4+TSDR-demethylated cells for cSCC risk using receiver operator characteristic analysis. The areas under the curve were 0.68 for % FOXP3+ CD4+ T cells and 0.70 for % CD4+ TSDR-demethylated cells, respectively.

## Conclusion

This is the first study to confirm stability of immune phenotype of peripheral blood leukocytes in long-term KTRs during follow-up, and the persistence of a different immune phenotype in KTRs with and without cSCC. KTRs that had previously developed a cSCC continued to have a higher proportion of FOXP3+ CD4+ T-lymphocytes and a reduced number of CD8+ Cm T cells than those who were cSCC free, reinforcing the observations made in the initial study cohort (11). The high death rate during the recruitment interval of the two cohorts emphasizes the increasing observation of the impact of death with a functioning graft on long-term outcome (20).

An important finding of this study is the consistency of key components of the immune phenotype over time in chronically immunosuppressed patients. This strengthens the potential use of immune phenotype as a biomarker of cancer risk posttransplantation. Importantly, the proportion of FOXP3+ CD4+ cells remains stable, making it suitable for use as a biomarker for cancer posttransplantation. We have assumed that the number of TSDR-demethylated cells is likely to remain consistent in line with the total Treg numbers. It would be interesting to prospectively reassess the stability of TSDR demethylation in Tregs, in this or a similar cohort of transplant patients with cSCC to confirm this assumption in the future. We found a large variation in the proportion of CD8 and CD4 subsets, as well as CD8+ CD28− regulatory cells, which we mainly attribute to aging (19).

This study demonstrates that patients who have previously developed a cSCC have increased numbers of FOXP3+ CD4+ T cells, which may prove to be a useful marker of skin cancer in transplant recipients. This observation is supported by several other studies, as well as FOXP3 being linked to cancer risk and outcome in the general population (11,21)–(24). It is important to recognize that there are other potential etiological factors, which could interact with the host immune phenotype such as a potential effect of chronic beta-human papilloma virus colonization (25). While studies on mice have shown FOXP3 to be a direct marker of Tregs, FOXP3 has also been shown to be up-regulated upon activation of human non-Treg effector cells (16,17). The up-regulation of FOXP3 upon activation of effector cells in the human results in an over-representation of Tregs, particularly in transplant patients due to chronic immune stimulation. Utilization of the TSDR methylation status allows identification of true Tregs (18). We have shown that TSDR-demethylated CD4+ lymphocytes amplify the association of higher proportions of Tregs in KTRs with cSCC compared to those without cancer. Interestingly, we found that the proportion of FOXP3+ cells was less than the proportion of TSDR-demethylated CD4+ cells. One previous study utilizing a ratio of FOXP3+ to TSDR-demethylated CD4+ cells showed that pediatric liver transplant recipients taking a CNI had a ratio <1 compared to those on a CNI-free regimen (26). Here, we replicate these findings, with patients on CyA having a FOXP3:TSDR ratio of <1, significantly lower than those on a CNI-free regimen. This together with the other published data suggest that Tregs from patients on CNI have impaired transcription of the FOXP3 gene, while TSDR FOXP3 demethylation remains to identify “true” Tregs (18).

Immune phenotype may prove to be a valuable biomarker for the identification of cancer posttransplantation. Stability of the immune phenotype demonstrated in this study, strengthens its value as a useful clinical tool. Elevated circulating Treg levels have been shown to be associated with cancers in both immunosuppressed and nonimmunosuppressed individuals. TSDR methylation analysis allows for a precise method of identification of true Treg numbers. TSDR-demethylated lymphocytes may prove to be a valuable indicator of cancer posttransplantation.

## Abbreviations

CNI: calcineurin inhibitor
cSCC: cutaneous squamous cell carcinoma
KTR: kidney transplant recipient
NK: natural killer
PBMCs: peripheral blood mononuclear cells
Treg: regulatory T cell
TSDR: Treg-specific demethylated region
